# The Elusive SLIPPERS Syndrome (Supratentorial Lymphocytic Inflammation with Parenchymal Perivascular Enhancement Responsive to Steroids): A Case Report and Literature Review

**DOI:** 10.2147/IMCRJ.S411204

**Published:** 2023-06-10

**Authors:** Adnan Khan, Muhammad Mohsin Khan, Issam A Al-Bozom, Younis Baregzai, Wanis Ibrahim, Firas Hammadi

**Affiliations:** 1 Department of Neurosurgery, Hamad General Hospital, Hamad Medical Corporation, Doha, Qatar; 2 Department of Laboratory Medicine and Pathology, Hamad Medical Corporation, Doha, Qatar; 3 Department of Medicine, Hamad General Hospital, Hamad Medical Corporation, Doha, Qatar

**Keywords:** CLIPPERS, SLIPPERS, corticosteroids, neuroinflammation, perivascular infiltration

## Abstract

**Background:**

In 2015, the term “SLIPPERS” was created to refer to a rare type of encephalomyelitis called CLIPPERS syndrome that affects the pons and sometimes other nearby structures, but in this case, it primarily affects the supratentorial region. This variation of the condition is responsive to treatment with steroids.

**Case Description:**

We report the case of a patient who presented with seizures and visual field deficit and had typical radiological and histopathological characteristics of SLIPPERS syndrome.

**Conclusion:**

Although the literature is inundated with CLIPPERS syndrome, its supratentorial variant is extremely rare. To our knowledge, this is fourth case of SLIPPERS syndrome to be reported in literature and serves to enhance clinicopathological understanding of this elusive entity.

A 21-year-old man with no comorbidities had presented to the ED with a history of two episodes of generalized tonic colonic seizures. He was vitally stable, with Glasgow Coma Score (GCS) 15 and neurologically intact. Routine laboratory investigations including complete blood count, urea and electrolytes and coagulation profiles were within normal limits. Electroencephalography was unremarkable. Brain computed tomography (CT) was reported normal (Figure 1A). Brain magnetic resonance imaging (MRI) without contrast showed small patches of right occipital subcortical hyperintensity on T2WI and FLAIR sequences (Figure 1B and C). The patient was assessed by a neurologist and subsequently discharged on anti-epileptic medications. A week later, the patient presented again with history of multiple incidents of colliding his head with objects while walking. A formal visual field assessment by Humphrey perimetry showed left homonymous hemianopsia. He was admitted to inpatients for further workup. MRI head with contrast (Figure 1D and E) was done and showed right parieto-occipital parasagittal subcortical and periventricular areas of T2W/FLAIR hyperintensity extending to involve right side of splenium of corpus callosum and demonstrating a patchy and nodular branching pattern of enhancement on T1WI with contrast (Figure 1F–H). Radiological features were suggestive of a diagnosis of infective and/or inflammatory process. Laboratory investigations including C-reactive protein, the Erythrocyte Sedimentation Rate (ESR), vitamin B12, Angiotensin Converting Enzyme level, and liver, thyroid and renal function tests were normal. Workup for autoimmune pathology including antinuclear antibody (ANA), anti-double-stranded DNA antibody (anti-ds DNA) and antineutrophil cytoplasmic antibody (ANCA) were within normal limits. Serologic tests including syphilis, Human Immunodeficiency Virus (HIV), Hepatitis B (HBV) and Hepatitis C (HCV) viruses, Herpes Simplex Virus (HSV), schistosomiasis antibody, Toxoplasma antibody, *Borrelia burgdorferi*, anti-DNase B and anti-streptolysin titer were all negative. Lumbar puncture was done and analysis of the cerebrospinal fluid (CSF) revealed a white cell count of 43/µL mainly lymphocytic (99%). CSF biochemistry showed glucose 3.47 mmol/L and protein 0.45 g/L, no oligoclonal bands were detected. No organism was seen on microbiology. CSF cytology study did not detect malignant cells. Cultures of urine, blood and CSF were all negative.

**Figure 1:**
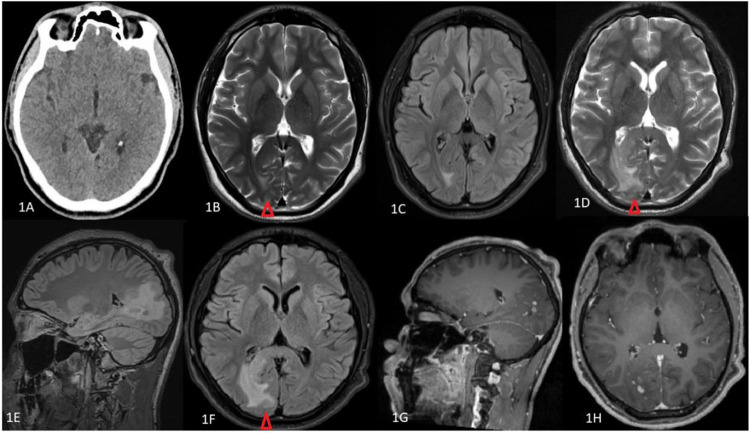
Radiological imaging. (**A**) CT of head without contrast (axial) does not show any abnormalities. (**B** and **C**) MRI of brain on 1st admission demonstrated small areas of right occipital subcortical patches of T2W (**B**) and FLAIR (**C**) bright signal. MRI of head on 2nd admission showed right parieto-occipital parasagittal subcortical and periventricular hyperintensity extending to the right side of splenium of corpus callosum on T2W axial (**D**), FLAIR sagittal (**E**) and FLAIR axial (**F**) sections (red arrowhead). MRI of head with contrast sagittal (**G**) and axial (**H)** sections revealing patchy nodular branching pattern of post-contrast enhancement of the hyperintense areas on T2/FLAIR. The enhancement is seen extending to the ependymal surface of occipital horn of right lateral ventricle.

The case created a diagnostic dilemma and was discussed in the Neurosurgery Multiple Disciplinary team (MDT) meeting which decided to proceed with biopsy of the lesion. Stereotactic biopsy of the right occipitoparietal lesion provided multiple samples of grayish white soft tissue for histopathological analysis which revealed fragments of gray and white matter heavily infiltrated by inflammatory cells with significant condensation of lymphocytes (CD45^+^) around the blood vessels (perivascular cuffing) (Figure 2A and B) as well as diffuse infiltration of the brain parenchyma by similar lymphocytes. Most of the lymphocytes were of T-lymphocytes (CD3^+^), (Figure 2C) of the T4 subtype (CD4^+^) (Figure 2D); while T8 subtype (CD8^+^) were much less (Figure 2E). Another population of B lymphocytes (CD20^+^, 79 A^+^) was seen at the periphery of the T cell population (Figure 2F). In addition, several clusters of foamy macrophages were seen within the lymphocytes (CD 163^+^) along with minor population of plasma cells (CD 138^+^). The brain showed reactive gliosis, vacuolar degeneration of the cytoplasm of the astrocytes as well as focal myelin loss and neuronal axonal degeneration. There was no evidence of fibrinoid necrosis of the blood vessels, granulomas, viral inclusions, fungal elements, or malignancy. In a follow up MDT meeting, the histopathologist and neuroradiologist agreed that the radiological and histopathological characteristics were compatible with SLIPPERS syndrome. The patient was started on IV methylprednisolone 1 g infusion administered over 3 hours daily for 5 days and was discharged on oral prednisolone 80 mg tapered over 6 weeks. On further follow up visit the patient showed improvement in his symptoms.

**Figure 2:**
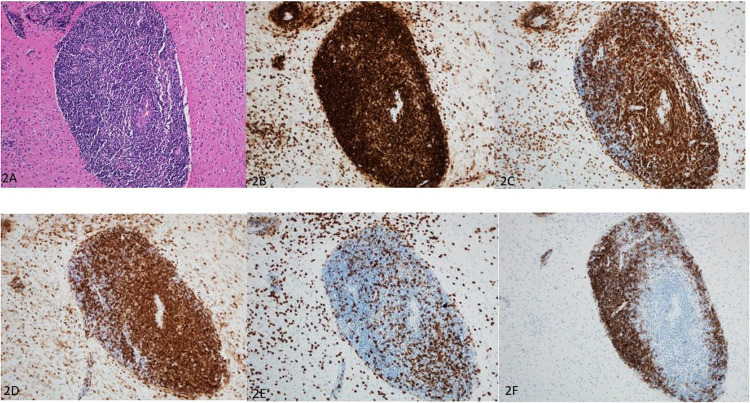
Histopathology. Microscopic examination of the biopsy specimen shows fragments of gray and white matter heavily infiltrated by inflammatory cells with significant condensation of lymphocytes (CD45^+^) around the blood vessels (perivascular cuffing) (**A** and **B**) as well as diffuse infiltration of the brain parenchyma by similar lymphocytes. Most of the lymphocytes are of T-lymphocytes (CD3^+^) (**C**) of the T4 subtype (CD4^+^) (**D**); while T8 subtype (CD8^+^) are much less (**E**). Another population of B lymphocytes (CD20^+^, 79 A^+^) was seen at the periphery of the T cell population (**F**). In addition, several clusters of foamy macrophages were seen within the lymphocytes (CD 163^+^) along with minor population of plasma cells (CD 138^+^).

The rarity of CLIPPERS as an encephalitis is underscored by the conspicuous involvement of pons.^1^ Pittock et al in 2010 were the first to describe CLIPPERS syndrome as a distinctive form of treatable encephalitis, emphasized the role of T lymphocytes in its pathogenesis and highlighted the response to corticosteroids.^2^ An immune-mediated and/or inflammatory pathogenesis is suggested because of evidence of response to immunosuppression with corticosteroids.^2^,^3^ The brainstem appears to be a favored target of immune attack, similar to that seen in neuro-Behcet’s disease.^4^ Clinical presentation of CLIPPERS syndrome varies widely but mainly incorporates symptoms related to the brainstem, cranial nerves and cerebellum, eg ataxia, vertigo, facial paresthesia, dysarthria and oculomotor abnormalities^2^,^5–9^ Furthermore, patients may also have symptoms due to involvement of spinal cord and/or long tracts including upper motor neuron (UMN) signs like spasticity and paresis, sensory deficits and sphincteric disturbances.^2^,^3^,^8–11^ Taieb et al found the clinical course of CLIPPERS syndrome to be relapsing and remitting without treatment in a study with long term follow up.^12^ Armand et al in 2015 introduced the term SLIPPERS syndrome when they reported 2 patients with a supratentorial pathology radiologically and pathologically indistinct from CLIPPERS syndrome. One patient had presented with recurrent seizures and the other with headache and hemiparesis.^13^ Horng et al in 2017 reported a third case of SLIPPERS syndrome with isolated cognitive dysfunction.^14^ The patient we have reported had presented with seizures and visual field defect.

CLIPPERS syndrome is characterized on MRI by punctate or nodular gadolinium enhancing lesions in the pons with variable involvement of the surrounding structures. Cranial extension of these lesions to encompass the thalamus, basal ganglia, corpus callosum, cerebral white matter, and caudal involvement of medulla oblongata as well as cervical and thoracic spinal cord has also been reported in the literature.^2^,^6^,^9^,^12^,^15^ Interestingly, the more distant the lesion from the pons, the less intense they become.^2^,^11^,^14^ MRI of the brain with contrast of our patient (Figure 1) showed radiological features reminiscent of the acronym PEPPERING. Since the lesions occurred supratentorially, our case report furthers evidence for SLIPPERS syndrome as an entity which shares radiological and clinicopathological similarities with the widely studied CLIPPERS syndrome. Pathological analysis of biopsied specimens from CLIPPERS syndrome patients reveals a remarkable perivascular, CD4-predominant T cell or lymphohistiocytic infiltrate,^2^,^8^,^12^ similar to our case. The course of CLIPPERS syndrome is marked by relapses and remission but generally it is not a progressive disease.^12^ Intravenous glucocorticoids administered in high dose for a short period of time followed by oral glucocorticoids appears to be the initial treatment of choice.^15^

We have identified a case of the exceedingly rare SLIPPERS syndrome, a variant of CLIPPERS syndrome, the diagnosis of which is based on typical radiological and histopathological features. The patient in our case report had unique presentation of visual field deficit and seizures. Being the fourth in the literature, our case adds to the scant literature available on SLIPPERS syndrome furthering the clinicopathological understanding this elusive entity.

## Abbreviations

ANA, Anti-nuclear antibody; ANCA, Anti-neutrophil cytoplasmic antibody; Anti-dsDNA, Anti-double stranded DNA; Anti-DNase B, Anti-Deoxyribonuclease B; CBC, Complete blood count; CLIPPERS, Chronic Lymphocytic Inflammation with Pontine Perivascular Enhancement Responsive to Steroids; CRP, C-Reactive protein; CSF, Cerebrospinal fluid; CT, Computed tomography; CNS, Central nervous system; ESR, Erythrocyte Sedimentation Rate; FLAIR, Fluid Attenuation Inversion Recovery; GCS, Glasgow Coma Score; HIV, Human Immunodeficiency Virus; HBV, Hepatitis B Virus; HCV, Hepatitis C Virus; HSV, Herpes Simplex Virus; MDT, Multiple Disciplinary Team; MRI, Magnetic resonance imaging; SLIPPERS, Supratentorial Lymphocyte Inflammation with Parenchymal Perivascular Enhancement Responsive to Steroids; T2W, T2 Weighted; T1W, T1 Weighted.

## Institutional Approval

Institutional approval was taken from Medical Research Council Qatar.
